# MRI based early Temporal Lobe Epilepsy detection using DGWO based optimized HAETN and Fuzzy-AAL Segmentation Framework (FASF)

**DOI:** 10.1371/journal.pone.0325126

**Published:** 2025-07-02

**Authors:** Hasim Khan, Ahmed Ibrahim Alutaibi, Ghanshyam G. Tejani, Sunil Kumar Sharma, Ahmad Raza Khan, Fuzail Ahmad, Seyed Jalaleddin Mousavirad

**Affiliations:** 1 Department of Mathematics, College of Science, Jazan University, Jazan, Kingdom of Saudi Arabia; 2 Department of Computer Engineering, College of Computer and Information Sciences, Majmaah University, Majmaah, Saudi Arabia; 3 Department of Research Analytics, Saveetha Dental College and Hospitals, Saveetha Institute of Medical and Technical Sciences, Saveetha University, Chennai, Tamil Nadu, India; 4 Department of Industrial Engineering and Management, Yuan Ze University, Taoyuan City, Taiwan; 5 Department of Information Systems, College of Computer and Information Sciences, Majmaah University, Majmaah, Saudi Arabia; 6 Information Technology Department, College of Computer and Information Sciences, Majmaah University, Majmaah, Saudi Arabia; 7 Respiratory Care Department, College of Applied Sciences, Almaarefa University, Diriya, Riyadh, Saudi Arabia; 8 Department of Computer and Electrical Engineering, Mid Sweden University, Sundsvall, Sweden; Bayer Crop Science United States: Bayer CropScience LP, UNITED STATES OF AMERICA

## Abstract

This work aims to promote early and accurate diagnosis of Temporal Lobe Epilepsy (TLE) by developing state-of-the-art deep learning techniques, with the goal of minimizing the consequences of epilepsy on individuals and society. Current approaches for TLE detection have drawbacks, including applicability to particular MRI sequences, moderate ability to determine the side of the onset zones, and weak cross-validation with different patient groups, which hampers their practical use. To overcome these difficulties, a new Hybrid Attention-Enhanced Transformer Network (HAETN) is introduced for early TLE diagnosis. This approach uses newly developed Fuzzy-AAL Segmentation Framework (FASF) which is a combination of Fuzzy Possibilistic C-Means (FPCM) algorithm for segmentation of tissue and AAL labelling for labelling of tissues. Furthermore, an effective feature selection method is proposed using the Dipper- grey wolf optimization (DGWO) algorithm to improve the performance of the proposed model. The performance of the proposed method is thoroughly assessed by accuracy, sensitivity, and F1-score. The performance of the suggested approach is evaluated on the Temporal Lobe Epilepsy-UNAM MRI Dataset, where it attains an accuracy of 98.61%, a sensitivity of 99.83%, and F1-score of 99.82%, indicating its efficiency and applicability in clinical practice.

## 1. Introduction

TLE is one of the most common types of epilepsy, occurring in approximately 1 in 2,000 individuals globally. It is responsible for roughly 60% of the cases of drug-resistant epilepsies [1]. TLE often results in major neurological and cognitive impairments that devastate the quality of life of the patient [2,3]. TLE is mostly attributed to predispositions inherited through genetics, brain infections, and traumatic injuries to the head, among other neurological disorders, such as hippocampal sclerosis. This could cause distortions to normal temporal lobe activities leading to unprovoked and recurrent seizures associated with TLE [4,5]. Tables 1 and 2 list the abbreviations and symbols utilized in the current result work, respectively.

**Table 1:** List of abbreviations.

Abbreviation	Description
TLE	Temporal Lobe Epilepsy
AD	Alzheimer’s disease
MRI	Magnetic Resonance Imaging
DGWO	Dipper- grey wolf optimization
3DCNN	Three-dimensional convolutional neural network
CNN	Convolutional neural networks
RD-fMRI	Resting-state functional MRI
WM	White matter
FPCM	Fuzzy Possibilistic C-Means
CSF	Cerebrospinal fluid
FASF	Fuzzy-AAL Segmentation Framework
PVL	Percentage volume loss
GW	Gray matter
HAETN	Hybrid Attention-Enhanced Transformer Network
DIR	Double inversion recovery
FDG	Fluorodeoxyglucose
fMRI	Functional MRI
Grad-CAM	Gradient-weighted class activation mapping
FC	Functional connectivity
ROI	Regions of interest
AAL	Automated Anatomical Labelling
PML	Percentage metabolic loss
DTO	Dipper-Throated Optimization
MPVA	Multivariate pattern analysis
SVM	Linear Support Vector Machine
LBP	Local Binary Patterns
VMHC	Voxel-mirrored homotopic connectivity
GWO	Grey Wolf Optimization
CBAM	Convolutional Block Attention Modules
GluCEST	Glutamate Chemical Exchange Saturation Transfer
Bi-LSTM	Bidirectional LSTM

**Table 2:** List of symbols.

Symbol	Symbol Legend	
	Name	Definition
X	Pixel/Voxel	A unit in an image, representing intensity at a specific location.
Min	Minimum Intensity	The lowest pixel intensity in an image.
Max	Maximum Intensity	The highest pixel intensity in an image.
$U_{IC}$	Fuzzy Membership	The degree to which pixel iii belongs to cluster kkk.
$V_{C}$	Cluster Center	The average intensity level of a cluster.
$D_{iC}$	Euclidean Distance	Distance between pixel iii and cluster center kkk.
M	Fuzziness Parameter	Controls the degree of fuzziness in clustering, usually m > 1m > 1m > 1.
i	Iteration Number	The count of iterations during optimization.
$J_{M}$	Objective Function	A function minimized to improve clustering.
n	Total Pixels/Voxels	The number of pixels or voxels in an image.
c	Number of Clusters	The total number of clusters (e.g., GM, WM, CSF).
$\alpha_{iC}$	Possibilistic Membership	Measures the likelihood of a voxel belonging to a cluster.
$\eta_{C}$	Tuning Parameter	Controls sensitivity in possibilistic clustering.
ε	Convergence Threshold	A predefined small value to stop iteration.
lbp	Local Binary Patterns	A texture descriptor capturing spatial patterns in images.
$m_{1}$	Mean Intensity	The average intensity of a segmented region.
$m_{2}$	Variance	Measures the spread of intensity values.
$m_{3}$	Skewness	Quantifies the asymmetry in intensity distribution.
area	Area	Total number of pixels in a segmented region.
perimeter	Perimeter	Length of the boundary of a segmented region.
eccentricity	Eccentricity	Describes elongation of a shape.
compactness	Compactness	Measures the regularity of a shape.
${BL}_{nd}(t)$	Bird Location	The position of a bird (feature vector) in Dipper-Throated Optimization.
BS(t+1)	Bird Speed	The velocity update of a bird during optimization.
$C_{1},\;C_{2},\;C_{3},C_{4},C_{5}$	Adaptive Parameters	Constants controlling feature selection in DGWO.
$r_{1}$	Random Number	A value in [0,1] used in optimization updates.
${\vec{x}}^{t}$	Wolf Position	The position of a solution in Grey Wolf Optimization.
$\vec{d}$	Prey Distance	Distance between a wolf and its target.
$\vec{a}$	Control Parameter	Adjusts exploration and exploitation in GWO.
$\vec{c}$	Inertia Weight	Balances exploration and exploitation in particle swarm optimization (PSO).
${\vec{c}}_{1},\;{\vec{c}}_{2},{\vec{c}}_{3}$	Acceleration Coefficients	Parameters in PSO that control cognitive and social behaviors.
α, β, δ, and ω	Leadership Positions	The best, second-best, and third-best solutions in GWO.
Fn	Fitness Function	Measures the quality of a solution.
${\vec{h}}_{t}$	Forward Hidden State	Hidden state of the forward LSTM at time step ttt
${\overset{\leftarrow }{h}}_{t}$	Backward Hidden State	Hidden state of the backward LSTM at time step ttt
$h_{t}$	Concatenated Hidden State	Combination of forward and backward hidden states at ttt
$\alpha_{t}$	Attention Weight	Weight assigned to each time step in the attention mechanism
$e_{t}$	Attention Score	Intermediate score for computing attention weight
$W_{a}$	Attention Weight Matrix	Learnable weight matrix in the attention mechanism
$b_{a}$	Attention Bias	Bias term for the attention score computation
v	Attention-Weighted Output	Summation of hidden states weighted by attention
Q,K,V	Query, Key, Value Matrices	Input representations in the multi-head attention mechanism
MultiHead (Q,K,V)	Multi-Head Attention	Operation combining multiple attention heads
$W^{o}$	Output Weight Matrix	Learnable weight matrix applied after multi-head attention
head	Number of Attention Heads	Total number of parallel attention heads
X	Input Embeddings	Embedding representation of the input sequence
P	Positional Encoding	Encodes positional information in transformer models
O	Transformer Output	Output from the transformer encoder layer
FFN	Feed-Forward Network	A position-wise feedforward neural network in transformers
LayerNorm(.)	Layer Normalization	Normalization layer used in transformers
$y_{i,j,k}$	Depthwise Convolution Output	Output of depthwise convolution at position (i,j)(i,j)(i,j) for channel kkk
$w_{mnk}$	Depthwise Convolution Filter	Filter applied in depthwise convolution
$y_{i,j,k}$	Pointwise Convolution Output	Output of pointwise convolution at position (i,j)(i,j)(i,j) for channel lll
$w_{kl}$	Pointwise Convolution Filter	1 × 1 convolution filter mapping depthwise convolution output
$M_{c}(X)$	Channel Attention Output	Output of channel attention mechanism
$M_{s}(X)$	Spatial Attention Output	Output of spatial attention mechanism
σ	Sigmoid Activation	Activation function applied in attention mechanisms
Conv2D	2D Convolution	Convolution operation applied in spatial attention
AvgPool(X)	Average Pooling	Computes global average pooling of XXX
MaxPool(X)	Max Pooling	Computes global max pooling of XXX
$X^{\prime }$	Attention-Weighted Feature Map	Final feature map after applying both channel and spatial attention
$W_{fc}$	Fully Connected Layer Weights	Weight matrix for the fully connected classification layer
$B_{fc}$	Fully Connected Layer Bias	Bias term for the fully connected classification layer
$P_{final}$	Final Class Probability	Softmax probability distribution for classification
Softmax	Softmax Function	Converts logits into a probability distribution

TLE is best treated and managed if diagnosed early and accurately. Previous approaches of TLE diagnosis involve clinical evaluation, followed by a qualitative analysis of MRI scans where time is greatly consumed and the results are prone to human errors [6,7]. Moreover, the minute anomalies signifying TLE often slip through routine assessment procedures. Therefore, it becomes critical to design more efficient, reliable, and automated detection tools that will aid physicians in the early identification of TLE [8]. Early diagnosis shall thus drastically improve the prognosis for the patients. In this regard, thorough interventions can be made to control the progression of the disorder, thus improving quality of life [9,10].

In the last few years, DL has been extended to automatically improve the diagnostic capabilities of medical imaging, especially in the neuroimaging domain. CNNs are the most applicable deep learning models in image analysis since they allow the detection of many patterns and features within medical images [11,12]. These models have shown great effectiveness in diagnosing different neurological disorders including Alzheimer’s disease, multiple sclerosis, and brain tumours [13]. However, their application in the context of TLE detection remains underexplored. Current MRI based approaches for TLE detection include CNN models for distinguishing between TLE, Alzheimer’s and normal control subjects based on T1w images, 3DCNNs trained on RS-fMRI for seizure prognosis and automated classification of hippocampal pathology lateralization based on multimodal MRI data [14,15]. These methods have been found useful but they have some drawbacks such as low specificity, high computational costs, data type dependency and low transferability across different patients. This work aims at addressing this gap by developing a new DL model for TLE identification using structural MRI scans as a quantitative and objective diagnostic approach.

The major contribution of the work includes:

- To detect TLE based on the Temporal Lobe Epilepsy-UNAM MRI Dataset.
- To introduce novel FASF which is an integration of the FPCM algorithm and AAL labelling to enhance tissue segmentation, achieving more accurate delineation of brain regions for TLE detection.
- To implement the advanced pre-processing techniques, including skull stripping, bias field correction, min-max normalization, and median filtering, to ensure high-quality input MRI images.
- To develop an advanced novel DGWO algorithm for selecting the most relevant features, reducing computational complexity, and enhancing model performance.
- To implement HAETN deep learning model that integrates attention mechanisms and transformer-based architectures to achieve superior accuracy, sensitivity, and robustness in TLE detection

The organization of the work: Section 2 contains review of literature, section 3 presents the proposed methodology in detail followed by simulation result and discussion in section 4, and finally the suggested work concludes in section 5.

## 2. Related work

In 2023, Chang *et al.* [16] suggested the use of CNN algorithm for the classification of TLE, AD and healthy control subjects using T1-weighted MRI scans. Further, they suggest using feature visualization methodologies to determine the parts of the brain that the CNN uses to distinguish between these diseases.

In 2022, Luckett *et al*. [17] proposed to use a 3DCNN that is trained with RS-fMRI data from the healthy controls with synthetic changes to the regions of interest for predicting the side of the TLE patient’s seizure onset. Further, they suggest applying Grad-CAM to determine the areas of the brain that provide the most discriminative information regarding the seizure onset zones.

In 2021, Caldairou *et al*. [18] designed an MRI based fully automated classifier for lateralizing covert hippocampal pathology in TLE patients based on T1, T2 and FLAIR/T1 features. The classifier is established on MRI data of patients with histologically confirmed HS and is expected to yield a higher lateralization than electroclinical data, including the side of surgery. The model’s performance is also evaluated in TLE cohorts outside the model, that is, in independent TLE cohorts.

In 2021, Beheshti *et al*. [19] suggested the analysis of the DIR data in combination with machine learning to differentiate between normal controls and epileptic patients as well as to determine the focus side in MRI-negative PET-positive TLE patients. They use whole-brain DIR data from participants who were scanned with high-resolution structural MRI and DIR to train a linear support-vector machine model.

In 2022, Aslam *et al*. [20] utilized volumetric MRI and 18F FDG PET to non-invasively diagnose or rule out TLE based on statistically generated threshold of asymmetry in these imaging studies. They plan to do so using PVL from amygdalohippocampal volumetry and PML from PET in order to distinguish TLE patients from extra TLE patients.

In 2021, Qu *et al*. [21] suggested the use of 3D-CNN framework derived from ResNet to detect mesial TLE in T2-FLAIR MRI images. The framework is to find the symmetrical differences of the corresponding brain areas, where the inputs are the symmetrical cubes. The proposed 3D-CNN is then compared with radiomics algorithms and visual assessment to demonstrate its potential for accurate and efficient diagnosis of MTLE, and could be used as a CAD system for epilepsy patients.

In 2021, Sherman *et al*. [22] proposed a clinical automated quantitative MRI measurement within statistical models to identify surgery outcomes for TLE patients. This entails employing pre-surgical T1-weighted MRI volumetric measurements derived from NeuroQuant to estimate the probability of seizure freedom and an Engel score of I at the time of surgery. The proposed study will also improve the prediction model and the role of volumetric data to include cortical volume loss, both focal and diffuse changes outside the surgical area affecting the seizure outcomes.

In 2021, Fu *et al*. [23] suggested that resting state fMRI and network-based connectivity analysis should be employed to compare the functional connectivity between MTLE and BECT. They argue that reduced functional connectivity in MTLE particularly between cortical networks and subcortical structures such as the hippocampus means the network is low efficiency and associated with poor prognosis. On the other hand, hyperconnectivity in BECT might be a compensatory process, which could be the reason for the better outcome. The findings of this analysis will seek to explain the differences in the brain network patterns of different types of epilepsy.

In 2021, Shi *et al.* [24] suggested that one should explore alterations in functional homotopy and FC in the whole brain in TLE. It also intends to determine which brain regions are important for classification through fMRI. The study applies VMHC and MVPA to determine areas in the brain affected by TLE and their correlation with the neuropsychological tests.

In 2021, Hadar *et al*. [25] proposed a three-dimensional GluCEST imaging for analysing brain glutamate networks in patients with no lesional TLE. This method is an advancement from previous single-slice glutamate imaging, allowing for more comprehensive spatial analysis. It aims to lateralize seizure onset in MRI-negative, no lesional TLE patients by detecting increased ipsilateral GluCEST signal in the hippocampus. Table 3 represents the comparison of existing techniques.

**Table 3:** Comparison of existing techniques.

Authors, Year	Techniques	Databases	Advantages	Disadvantages	Outcomes
Chang et al, 2023	CNN	T1-weighted MRI	High accuracy in identifying TLE; high precision and recall	Only 47% of cohort deemed lesional on MRI; model dependent on MRI data	Accuracy: 90.45%, Precision: 0.86, Recall: 0.86
Luckett et al, 2022	3DCNN	RS-fMRI data from 2132 controls, 32 TLE patients	High classification accuracy for TLE hemisphere localization	Relatively small sample size for TLE patients	Accuracy: 90.6%
Caldairou et al, 2021	Surface-based Linear Discriminant Classifier	T1, T2-weighted, FLAIR MRI of 60 TLE patients	High lateralization accuracy, effective in MRI-negative cases	Relies on MRI features, may miss pathology	Lateralization accuracy: 93%, AUC: 0.95
Beheshti et al, 2021	SVM	Whole-brain DIR MRI data from 63 participants	High accuracy in discriminating between left, right TLE, and HC	Limited sample size	Overall Accuracy: 87.30%
Aslam et al, 2022	Statistical Analysis (ROC, Volumetric Measurement)	Volumetric MRI & FDG PET data	Effective for non-invasive TLE detection in MRI-negative cases	Limited patient sample	Sensitivity: 88.89%
Qu et al, 2021	3D-CNN	T2-FLAIR MRI data from 15 MTLE patients	Utilizes 3D-CNN to incorporate structural data and spatial relationships	Small patient sample	Accuracy: 93.02%
Sherman et al, 2021	Quantitative MRI Measurements & Statistical Models	Volumetric T1-weighted MRI from 435 patients	Enhances prediction accuracy for surgical outcomes	Not always applicable in routine clinical settings	P-value: 0.02
Fu et al, 2021	Network-based Connectivity Analysis (fMRI, ICA)	Resting-state fMRI (14 MTLE, 12 BECT, 16 HC)	Provides insights into cortical-subcortical functional connectivity patterns	Small sample size, does not address all subtypes of epilepsy	P-value: 0.951
Shi et al, 2021	Functional Homotopy & Functional Connectivity (fMRI)	fMRI data from TLE patients and HC	Provides a deep dive into altered brain connectivity in TLE	Limited sample size and homogeneity of patients	Accuracy: 68.49%, Sensitivity: 66.67%, Specificity: 70.27%;
Hadar et al, 2021	3D GluCEST	4 MRI-negative TLE patients	Advanced 3D imaging of glutamate activity, better seizure localization	Small cohort size, MRI-negative cases	P-value: 0.048

## 3. Proposed methodology

**Fig 1:**
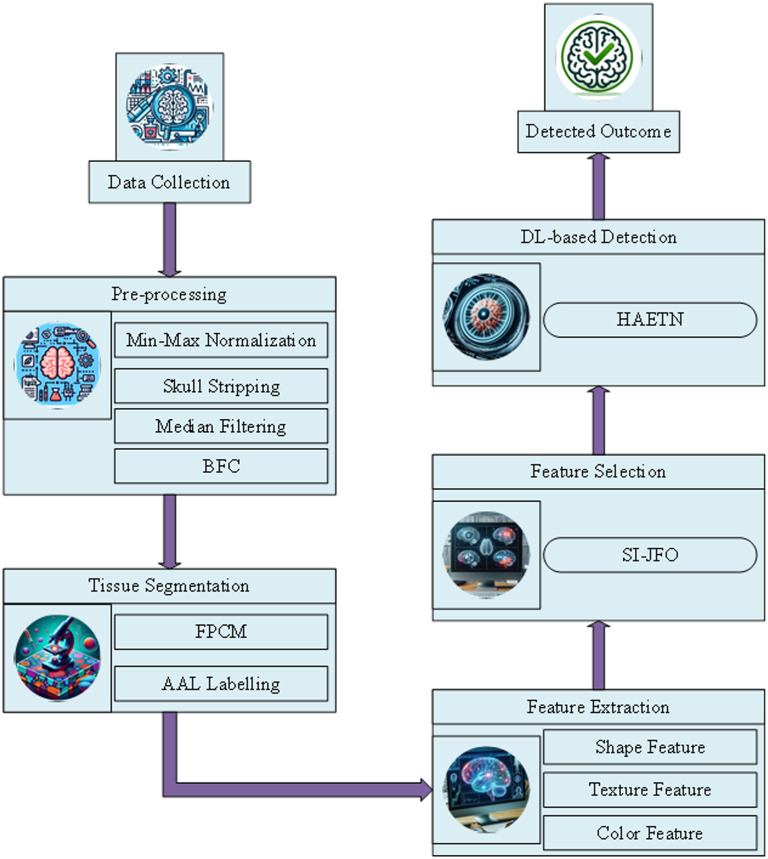
Architecture of the proposed approach.

This work presents a new method for diagnosing TLE, a type of epilepsy that originates in the temporal region of the brain. The detection process starts from obtaining raw MRI images from the Temporal Lobe Epilepsy-UNAM MRI Dataset after which the images undergo rigorous pre-processing stages including skull stripping, bias field correction, min-max normalization and median filtering to provide improved input image for the subsequent steps. For tissue segmentation, the newly developed FASF which integrates FPCM algorithm and AAL labelling to improve the segmentation of different brain regions. After that, texture, shape and colour feature techniques are used for feature extraction from the segmented images, which offer a complete description of essential features that can be used to achieve accurate TLE identification. To select optimal features, the DGWO is used to improve feature selection, minimize computational burden, and improve model performance. Last, the detection of TLE is done through the newly developed HAETN, a deep learning model that combines attention and transformer-based structures to provide higher accuracy, sensitivity, and robustness. This integrated approach is a major step forward in improving the detection of TLE and can overcome the existing methods to provide better diagnostic and clinical results. The architecture of the proposed approach is represented in Fig 1.

**Data collection and ethical consideration:** The suggested approach is evaluated based on the Temporal Lobe Epilepsy-UNAM MRI Dataset (https://openneuro.org/datasets/ds004469/versions/1.1.3/download) This dataset contains resting-state fMRI and task-based fMRI data specifically designed for evaluating working memory. It includes imaging data from both patients diagnosed with TLE and healthy control participants. This comprehensive dataset supports studies on memory function and neural dynamics in TLE and healthy populations.

**Ethical considerations:** Secondary analysis was performed on this publicly available data set. The Temporal Lobe Epilepsy-UNAM MRI Dataset is completely anonymized and had been made available within the bounds of ethics for data sharing. Thus, the dataset was used only for scientific research and had no information revealing the identities of subjects. There is no further need for ethics approval or informed consent because it is a retrospective analysis of anonymous data drawn from an open-access source. This research conformed to the code of ethics, as laid down by the institutional review boards and other worthy professional organizations.

### 3.1. Pre-processing

Normally, pre-processing is applied to attain noise-free data from the raw images. In case of brain MRI, the complex structures need to be pre-processed to enhance image quality and highlight the significant areas. It includes:

#### 3.1.1. Skull stripping.

Skull stripping is a process of eradicating all the features other than the brain tissue from the MRI or CT scan image. In TLE detection, skull stripping is required because it separates the brain from other structures. This enables deep learning models to concentrate on the regions of the brain that are most useful in identifying the abnormalities linked with epilepsy, including the temporal lobe lesions while excluding the non-brain tissues. When skull stripping is done, the image only has the brain region that is void of the skull and soft tissue artefacts [26]. This cleaned-up image benefits the detection algorithms as it minimizes the noise, increases the accuracy of brain region segmentation, and makes it easier to identify areas related to epilepsy.

#### 3.1.2. Bias Field Correction (BFC).

Bias field correction serves to promote image quality. Most images have intensity in-homogeneity problems due to bias fields. Bias field signals are low-frequency signals that attenuate high-frequency information and thus degrade image quality [27]. Bias field correction is, therefore, an attempt to remedy this by applying energy-minimization operations. Bias field correction proceeds in two steps in which images are decomposed into two multiplicative components. The first component is the estimation of the bias field, followed by the correction of the bias field. These two components are optimized using energy minimization methods.

BFC is required in TLE identification since MRI images are susceptible to intensity inhomogeneity resulting from variations in the magnetic field. These inhomogeneities can also affect the tissue contrast which in turn affects the ability of deep learning models to accurately identify abnormalities such as lesion or structural change in the brain [28]. After BFC, the image intensity is more uniform throughout the brain because the intensity variations due to the MRI scanner’s magnetic field are minimized. This results in improved definition and less ambiguity in the depiction of the brain tissues, which in turn helps the model to identify corresponding features for TLE diagnosis with more precision.

#### 3.1.3. Min-Max-Score Normalization.

Min-Max-Score Normalization is a preprocessing technique used to scale image pixel intensities to a fixed range, typically between 0 and 1. This is achieved using the formula:


$$Normalized\;value=\frac{Pixel\;value-Min}{Max-Min}$$ (1)

where Min and Max are the minimum and maximum intensity values in the image.

Min-Max-Score Normalization is important in the detection of TLE as it ensures that all the pixel intensities are scaled consistently, so all the features contribute proportionately to the learning process and prevents dominance by higher intensity ranges. This improves the accuracy of the model, accelerates the convergence during training, and also enhances the contrast uniformity of the image. Rescaling pixel values to a defined range (e.g., 0–1) after normalization in order to optimize the image for effective feature extraction and analysis by the deep learning model [29].

#### 3.1.4. Median Filter.

**Fig 2:**
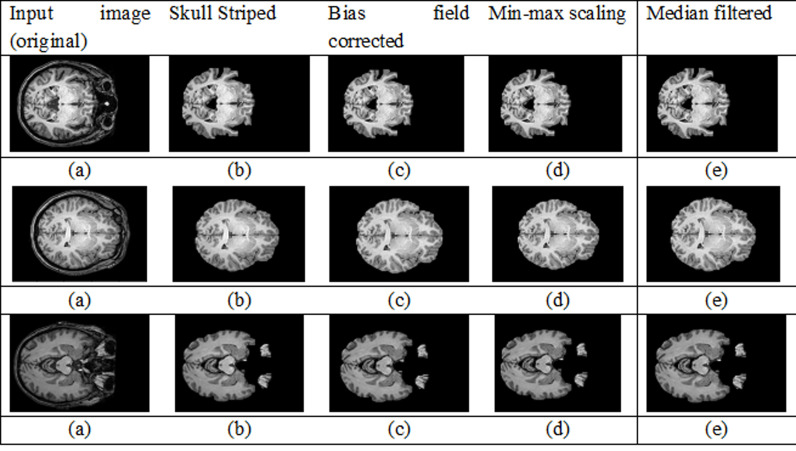
Illustration of the MRI image preprocessing and further segmentation process. **(a)** Median filtered images used to enhance tissue contrast along with suppressing noise. **(b)** Fuzzy Possibilistic C-Means (FPCM) clustering outcomes with the segmented brain regions and corresponding distinct tissue classes. **(c)** Anatomical labeling by AAL-or Automatic Anatomical Labeling-atlas with matching segmented regions to known brain structures for its anatomical localization and interpretation.

The Median Filter is a non-linear technique in image processing and it eliminates noises in MRI images by replacing the central pixel of a given window with the median of the neighbouring pixels. This effectively eliminates salt-and-pepper noise and small variations of the intensity while keeping edges and other structures. In the detection of TLE, the Median Filter is essential in enhancing the quality of the image, since it removes noise without blurring the features that are critical for proper analysis. After filtering, the MRI image becomes smoother with minimized noise and preserved key anatomical details, allowing the deep learning model to extract more reliable features. Fig 2 presents the MRI images at various pre-processing stages. Fig 2 shows some sample MRI images and the different stages of pre-processing. Each row is for a different MRI subject, highlighting improvements at every step.

- Column one contains the input images in their original form, including noise, skull artifacts, and intensity inhomogeneities (Fig 2a).
- Column two (Fig 2b) applies skull stripping to remove non-brain tissues, retaining the brain area for critical analysis.
- Column three (Fig 2c) shows bias field-corrected images in which intensity non-uniformity due to scanner flaws or inconsistencies in the magnetic field is corrected, creating a more homogeneous appearance across brain tissues.
- Then Column four (Fig 2d) displays a min-max scaling of the images whereby pixel intensities are normalized within a specified range that allows for faster as well as more stable convergence of the model during training.
- Then Column five (Fig 2e) represents the median-filtered images smoothed further to suppress any residual noise but maintain important brain structures and edges.

This pre-processing step sequence is very important in making sure that the input data is clean, consistent, and optimally adapted for reliable segmentation and classification within the next stages of the proposed framework.

### 3.2. Segmentation phase via Fuzzy-AAL Segmentation Framework (FASF)

**Fig 3:**
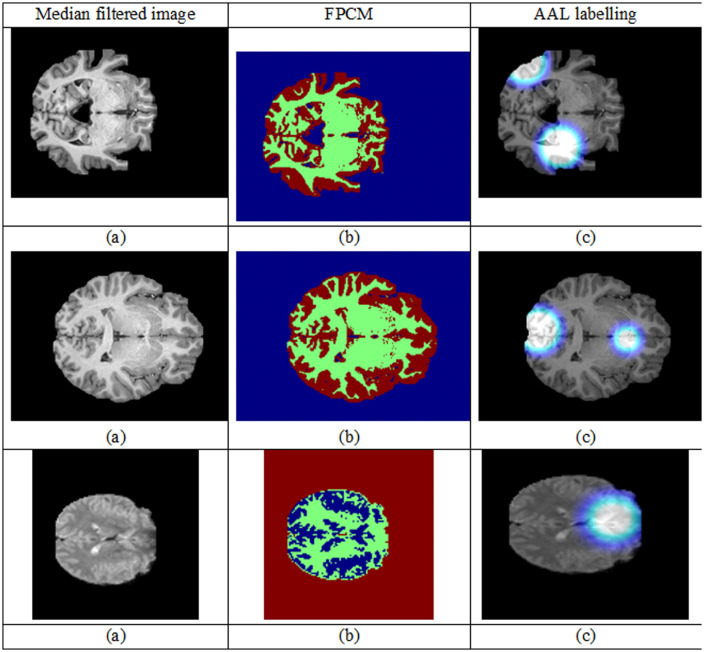
Visualization of model attention via Gradient-weighted Class Activation Mapping (Grad-CAM). **(a)** Original MRI slices as feeds into the deep learning model. **(b)** Grad-CAM heatmaps depicting the most influential zones contributing to model’s decision making, where warmer colors mean stronger activations. **(c)** Superimposed pictures of the Grad-CAM heatmap with the original MRIs, allowing the interpretation of the model’s predictions in relation to the anatomy.

One of the best algorithms for segmenting brain tissues is the extension of the original FCM known as the FPCM, adding the concept of possibilistic membership to solve some of the constraints of the original FCM, in coping with noise and outliers often found in medical imagery like MRI. FPCM combines the merits of fuzzy clustering (soft membership) with probabilistic clustering (robustness against outliers), which makes it suitable for the complicated nature of MRI data. FPCM is the technique used in medical image segmentation, which is especially relevant for MRI scans, where fuzzy and possibilistic clustering combine to improve medical image segmentation. The fuzzy component allows every voxel to belong to different tissue types with different membership degrees and supports overlapping or gradually transitioning tissues, and the possibilistic component improves robustness by dealing with noisy data and outliers. The fuzzy membership represents the degree of association of each voxel with its tissue type, based on similarity in intensity, and the possibilistic membership lowers the influence of the outliers. The objective function is iterated until it converges, and the mean intensity values for each tissue type (GM, WM, CSF) are indicated by the cluster centres. This results in high-quality segmentation so that ROI are delineated accurately, which is crucial for the detection of diseases like TLE from MRI data. Fuzzy-AAL Segmentation Framework (FASF) represents segmentation results on brain MRI scans in Fig 3. Each row signifies a patient MRI, thereby indicating the resilience of the pre-processing and segmentation pipeline against different sets of anatomical structures and intensity variations.

- First column entries (Median-filtered images, Fig 3a) show considerable noise reduction in MRI scans while retaining the fine anatomical details. The Median Filter is applied to buff off salt-and-pepper noise and random noise artifacts that do exhibit major areas of interest like the hippocampus or the temporal lobe structures, which become critical in the case of temporal lobe epilepsy (TLE) analysis. The cleaner images guarantee that the later segmentation processes get carried out on high-quality data, preserving the structural detail necessary for accurately localizing and delineating regions that harbor anomalies.
- The second column (FPCM segmentation results, Fig 3b) clearly displays the working of the Fuzzy Possibilistic C-Means Algorithm in segmenting the brain tissues into various regions that are based on intensities similarity. Here, Cyan pertains to intermediate brain tissues (gray matter); while the yellow indicates very intense tissues (such as white matter or perhaps lesion domains), Brown refers to probable lesions or specific anatomy variations, and dark blue highlights background or non-brain regions. Of particular note is the fact that the FPCM results evidenced clearer demarcation of brain compartments from non-brain elements thus giving an excellent intermediate representation more appropriate to clinical interpretation and further functional mapping.
- Third is the column showing the results for AAL Labelling, which applied the AAL atlas onto the pre-segmented images (Fig 3c). Mapping as such marks those areas that are very highly activated or very intense with White and possibly above functional significance. These are themselves colored Blue according to the various areas that have been anatomically labeled and which show the structural-functional mapping of the brain, whereas Red indicate particular anatomically or functionally relevant regions of interest (e.g., hippocampus, amygdala) and which are predominantly critical in studies of epilepsy. This segmentation provides the standard anatomical references to compare results across subjects as well as to enhance the interpretability of potential lesion localization by following up the tissue-level clustering achieved through FPCM.
- In general, the figure depicts a three-tiered segmentation capability of FASF: (1) suppressed noise imaging, (2) intensity-based clustering for tissue, and (3) anatomical labeling, all of which combine for accurate and interpretable detection of pathological features in TLE-related studies.

#### 3.2.1. Fuzzy possibilistic membership update.

During segmentation, membership functions $U_{IC}(fuzzy)$ and $\alpha_{IC}(possibilistic)$ are updated so that the degree of membership of a pixel i in a cluster C is established. The update of fuzzy membership $U_{IC}$ is defined by computing the distance between a pixel i and the centre of the cluster C, weighted with a fuzziness value M and it can be deliberated using the following Eq. (2),


$$U_{IC}=\frac{1}{\left(\sum_{K=1}^{c}{\left(\frac{D_{iK}}{D_{iC}}\right)}^{\frac{2}{M-1}}\right)}$$ (2)

where, $U_{IC}$ is the fuzzy membership of pixel i in a cluster C, $D_{iC}$ is the Euclidean distance between pixel i and the cluster centre C, M is the fuzziness parameter (usually M >1), c is the number of clusters (e.g., GM, WM, CSF). Outliers are accounted for by updating the possibilistic membership function $\alpha_{ic}$ and it can be arithmetically deliberated using the following Eq. (3),


$$\alpha_{ic}=\frac{1}{1+{\left(\frac{D_{iC}}{\eta_{C}}\right)}^{Q}}$$ (3)

It’s designated as $\alpha_{ic}$, which is the probability of a pixel i belonging to cluster C, D is the Euclidean distance between pixel i and cluster centre C, $\eta_{C}$ is a tuning parameter that controls the fuzziness of the cluster in probabilistic clustering, Q is a tuning parameter that controls the sensitivity of the membership.

The objective function $J_{M}$ combines fuzzy and possibilistic terms to reduce the distance between picture pixels and cluster centres by considering both types of memberships and it can be arithmetically deliberated using the following Eq. (4),


$$J_{M}=\sum_{i=1}^{n}\sum_{c=1}^{c}\left[U_{iC}^{M}D_{iC}^{2}+\alpha_{iC}^{Q}V_{iC}^{2}\right]$$ (4)

Here, the parameter $J_{M}$ is the objective function to be minimized, n is the total number of pixels or voxels in the MRI image, c is the number of tissue types or clusters (e.g., GM, WM, CSF), $U_{iC}$ is the fuzzy membership of voxel, i in cluster C, $\alpha_{iC}$ is the possible membership of Voxel i in cluster C, $D_{iC}$ is the distance between the voxel i and cluster centre C, $V_{iC}$ is the possibilistic distance (a measure of how far the voxel i is from the cluster centre).

**Cluster Centre Update:** $V_{C}$ is the average intensity level of the corresponding tissue types. The centres are updated iteratively by the weighted membership of the pixels. Moreover, the cluster centre updating has been mathematically deliberated in the following Eq. (5),


$$V_{C}=\frac{\sum_{i=1}^{n}\left(\alpha_{iC}^{Q}U_{iC}^{M}.X_{i}\right)}{\sum_{i=1}^{n}\alpha_{iC}^{Q}U_{iC}^{M}}$$ (5)

Here, $V_{C}$ is the updated cluster centre of tissue type C, $X_{i}$ is the intensity value of voxel i, $U_{iC}$ and $\alpha_{iC}$ are fuzzy and possibilistic membership values of voxel i in cluster C.

**Stopping Criteria:** The iteration is reiterated till convergence, and usually, it is measured by the variation of objective function between two successive iterations, thus it can be mathematically given in Eq. (6),


$$\left\|J_{M}(K+1)-J_{M}(K)\right\|>\varepsilon$$ (6)

Here, the following parameters $J_{M}(K+1)$ and $J_{M}(K)$ were the objective functions over the iterations (K+1), as well as Kcorrespondingly and ε is the lowest threshold rate which indicates the level of convergence.

**Segmentation Results:** After convergence, each voxel is assigned to the tissue type with the highest membership value, which may be fuzzy or possibilistic. Segmented tissue maps such as GM, WM, and CSF can be obtained by using Eq. (7),


$$\begin{array}{c} \hat{Y_{i}}=\mathrm{\backslash arg}\max_{C}(U_{iC}.\alpha_{iC}) \end{array}$$ (7)

Here, the following parameter $\begin{array}{c} \hat{Y_{i}} \end{array}$ is the output segmented label for the voxel i. Moreover, the following parameters such as $U_{iC}$ and $\alpha_{iC}$ were the fuzzy and possibilistic memberships for voxel iin cluster C.

#### 3.2.2. AAL labelling.

The AAL procedure maps segmented brain areas to preset anatomical ROIs using the AAL template. The transformation process can be described as follows in a numerical form by using the following Eq. (8),


$$t(X,Y,Z)=\mathrm{\backslash arg}\max_{i}(r_{i}(X^{\prime },Y^{\prime },Z^{\prime }))$$ (8)

Here,$\left(X^{\prime },Y^{\prime },Z^{\prime })=h(X,Y,Z\right)$

**Transformation Function** h(X,Y,Z): Affine or non-linear transformation is needed to align the segmented image coordinates (X,Y,Z) to the standardized space of the AAL atlas. This ensures that an accurate spatial correspondence between a patient’s MRI scan is achieved with predefined anatomical regions of interest in ROIs of the AAL template. This method, which is usually carried out using tools such as SPM or ANTs, standardizes the orientation and size of MRI data to allow precise mapping of segmented brain tissues into their correct regions, such as the temporal lobe, for further analysis and diagnosis.

**Region Mapping** $r_{i}(X^{\prime },Y^{\prime },Z^{\prime })$: This is an anatomically designated region in the AAL atlas, indexed by i. Moreover, the labelling function assigns each voxel to the ROI i that has the highest overlap or intensity correspondence after alignment.

**Final Mapping** t(X,Y,Z): Returns the anatomical label ifor each voxel in the transformed space. This will indicate the correctness of labelling regional divisions like gray matter, white matter, and CSF segmented into AAL-defined ROIs including the temporal lobe.

### 3.3. Feature extraction

The mathematical expressions for texture, color and shape features are manifested in Table 4.

**Table 4:** Mathematical formulas for texture, color, and shape features in the DL model.

Feature Type	Formula	Description
Texture	$lbp(x,y)=\sum_{N=0}^{p-1}\delta (i_{n}-i_{c}){\mathrm{.2}}^{N}$ (LBP)	Captures local spatial patterns
Color	$m_{1}=\frac{1}{n}\sum_{i=1}^{n}P_{i}$ (Mean)	Average intensity value of the color channel
	$m_{2}=\sqrt{\frac{1}{n}\sum_{i=1}^{n}(P_{i}}-m_{1}{)}^{2}$ (Variance)	Spread of intensities around the mean
	$m_{3}=\frac{1}{n}\sum_{i=1}^{n}(P_{i}-m_{1}{)}^{3}$ (Skewness)	Asymmetry of the intensity distribution
Shape	$area=\sum (X,Y)\in r^{1}$	Total number of pixels in the segmented region R
	$perimeter=\sum (X,Y)\in boundary(r{)}^{1}$	Length of the boundary of the segmented region R
	$eccentricity=\frac{C}{A},C=\sqrt{A^{2}-B^{2}}$	Describes elongation
	$compactness=\frac{perimeter^{2}}{area}$	Regularity of the shape

**Features from texture: Local Binary Patterns (LBPs):** LBP is critical in retrieving the textural features within the MRI images, thereby very useful in identifying the structural abnormalities that characterize TLE. LBP compares the intensity of a center pixel with the intensity of its neighbors, which it then encodes as a binary pattern. The patterns that result from this are histograms that portray local textures in grey matter, white matter, and cerebrospinal fluid. This strong representation is extremely helpful for the detection of minute textural changes that other approaches may miss.

**Color Features:** It summarizes the statistical features of the intensities of colors of an image, which is very crucial for MRI scans, especially when they have pseudo-color representations. The first moment corresponds to mean intensity, the second moment to the spread of intensities, and the third moment to the asymmetry in the distribution of intensity. These parameters characterize the tissue properties with a precise description of the abnormality in intensity patterns, suggesting epileptogenic areas in the temporal lobe.

**Shape features:** Shape features analyse the morphology of the segment regions in MRI images, depicting structural abnormalities in the temporal lobe. Area, perimeter, eccentricity, and compactness are the measures by which the size and shape and the border as well as the regularity of a region are quantified. An abnormal shape or abnormally large eccentricity of the segmented grey matter can point to degenerative changes associated with epilepsy. Form descriptors thus enhance the localization and identification capabilities of epileptic tissue by the DL model.

### 3.4. Feature selection using the proposed Dipper-Grey Wolf Optimization (DGWO)

The proposed DGWO algorithm is designed to enhance feature selection for temporal lobe epilepsy detection by selecting the most relevant features from texture, color, and shape attributes. By leveraging the combined strengths of DTO and GWO; DGWO achieves optimal feature selection while reducing computational complexity.

1) ***Dipper Throated Optimization***

The proposed DGWO takes into account the extracted texture, color and shape features. The DTO simulates the real process of identifying the locations and velocities of swimming and flying birds in order to locate food. Eq. (15) is used to update the position and speed of the swimming birds. The features that have the highest discriminating power (among texture, color as well as shape features) are prioritized based on the relevance to detection performance.


$${BL}_{nd}(t+1)={BL}_{best}(t)-C_{1}\cdot \left|C_{2}.{BL}_{best}(t)\right|-{BL}_{nd}(t)$$ (9)

where t represents the iteration number, ${BL}_{nd}(t)$ and ${BL}_{best}(t)$ denotes the normal location and best location of the bird, $C_{1}$ and $C_{2}$ are the adaptive values these values are changed based on the random values and iteration number during the optimization process. On the other side, perform the update of the flying bird’s location by the given Eqs (10) and (11).


$${BL}_{nd}(t+1)={BL}_{nd}(t)+BS(t+1)$$ (10)

$$BS(t+1)=C_{3}BS(t)+C_{4}r_{1}\left({BL}_{best}(t)-{BL}_{nd}(t)\right)+C_{5}r_{1}({BL}_{Gbest}-{BL}_{nd}(t))$$ (11)

where, BS(t+1) represents the updated speed of each bird, ${BL}_{Gbest}$ denotes the global best location, $C_{3}$ is the weight value, $r_{1}$ indicates the random number in [0,1], $C_{4}$ and $C_{5}$ are constants. Based on Eq. (17), the dynamic feature selection is accomplished by means of reducing the classification loss.

2) ***Grey Wolf optimization***

An old-fashioned metaheuristic algorithm called the Grey Wolf optimization (GWO) replicates the hunting habits of four grey wolves such as α, β, δ, and ω wolf in a pack. These wolves work together to find, locate, and encircle their prey. The algorithm continuously raises the goal of the solution space by using a mathematical model that imitates the foraging habits of a pack of grey wolves. The GWO algorithm’s main model is shown below.

There are two stages in the GWO algorithm: the siege stage and the seek-for-prey stage. The following Eqs (12) and (13) determines the siege phase:


$$\vec{d}=\left|\vec{c}\times {\vec{c_{p}}}^{t}-{\vec{x}}^{t}\right|$$ (12)

$${\vec{x}}^{\left(t+1\right)}={\vec{x}}^{t}-\vec{a}\times \vec{d}$$ (13)

where ${\vec{x}}^{t}$ represents the wolf position in iteration t, the position vector of prey is $\vec{d}$, $\vec{a}$ and $\vec{c}$ denote the coefficient vectors, which are determined in Eqs (14) and (15):


$$\vec{a}=2l\times r_{1}$$ (14)

$$\vec{c}=2\times r_{2}$$ (15)

Assume that throughout the hunting phase, alpha, beta, and delta will hunt and will know the potential for each position based on their experience. This can be expressed using Eq. (16) to Eq. (18), respectively.


$${\vec{d}}_{\alpha }=\left|{\vec{c}}_{1}\times {\vec{x}}_{\alpha }-\vec{x}\right|;{\vec{d}}_{\beta }=\left|{\vec{c}}_{2}\times {\vec{x}}_{\beta }-\vec{x}\right|;{\vec{d}}_{\delta }=\left|{\vec{c}}_{3}\times {\vec{x}}_{\delta }-\vec{x}\right|;$$ (16)

$${\vec{x}}_{1}={\vec{x}}_{\alpha }-{\vec{a}}_{1}\times {\vec{d}}_{\alpha };{\vec{x}}_{2}={\vec{x}}_{\beta }-{\vec{a}}_{2}\times {\vec{d}}_{\beta };{\vec{x}}_{3}={\vec{x}}_{\delta }-{\vec{a}}_{3}\times {\vec{d}}_{\delta }$$ (17)

$${\vec{x}}^{\left(t+1\right)}=\frac{{\vec{x}}_{1}+{\vec{x}}_{2}+{\vec{x}}_{3}}{3}$$ (18)

During the searching and attacking stage, wolves attack a victim if $\left|\vec{a}\right|<1$ and use a casually created vector $\vec{a\;}$within the range of [-2a, 2a]. To prevent local optimization, a random variable called c influences the decoy search. Thus, features are extracted based on the fitness function of the DGWO algorithm. Algorithm 1 presents the pseudocode of the hybrid DGWO algorithm. The adaptive tuning performance of DTO is utilized to explore the feature space, while the balance distribution of GWO is utilized to refine and exploit the selected features for enhanced classification performance.

***Algorithm 1: DGWO algorithm***

1. Initialize the location of the bird ${BL}_{i}(i=1,2,3,\cdots ,n)$ with size $n,{BL}_{i}(i=1,2,3,\cdots ,n)$

2.Fitness function Fn, $x_{n}$, $r_{1}$, $r_{2}$, $r_{3}$, R, $C_{1}$, $C_{2}$, $C_{3},$ $C_{4}$, $C_{5},\;t=1,$ and max iterations iter_max

                Fn=min(E)

Here, E is the classification error.

3. Evaluate fitness function Fn for each ${BL}_{i}$

4. Find the best bird ${BL}_{best}$

5.While t<iter_max  do

6.  for (i=1;i≤n) do

7.   If (R < 0.5) then

8.    Update the location of the grey wolf agents using:

9. ${\vec{d}}_{\alpha }=\left|{\vec{c}}_{1}\times {\vec{x}}_{\alpha }-\vec{x}\right|;{\vec{d}}_{\beta }=\left|{\vec{c}}_{2}\times {\vec{x}}_{\beta }-\vec{x}\right|;{\vec{d}}_{\delta }=\left|{\vec{c}}_{3}\times {\vec{x}}_{\delta }-\vec{x}\right|;$

10. else

11.   Update the speed of the flying bird using:

12. $BS(t+1)=C_{3}BS(t)+C_{4}r_{1}\left({BL}_{best}(t)-{BL}_{nd}(t)\right)+C_{5}r_{1}({BL}_{Gbest}-{BL}_{nd}(t))$

13.   Update the location of the swimming bird using:

14. ${BL}_{nd}(t+1)=r_{1}+z*r_{2}+(1-z)*r_{3}+BS(t+1)$

15. end for

16. Evaluate fitness function Fn for each ${\vec{BL}}_{i}$

17. Update $r_{1}$, $r_{2}$, $r_{3}$, R, $C_{1}$, $C_{2}$,

18. Find the best bird ${BL}_{best}$

19. Set ${BL}_{Gbest}={BL}_{best}$

20. Set t=t+1

21. end while

22. return ${BL}_{Gbest}$

### 3.5. Deep learning-based detection via HAETN

A new HAETN is introduced in this research work for accurate as well as early detection of the TLE. This HAETN model encapsulates the BiLSTM with Attention, Transformer-based models, Lightweight MobileNets, and CBAM, respectively. The sequence relationships existing in the images’ feature representations are taken up by the BiLSTM component, while the attention focuses on crucial areas regarding epileptic activity. The Transformer architectures extend capacity for training the model on ultimately more global dependencies and contextual information across feature maps via multi-head self-attention and positional encoding. The lightweight MobileNets contribute to efficient extraction of features, thus allowing the model to deploy in a scenario of constraint resources. The CBAM applies channel and spatial attention to only attend to relevant features that improve the detection’s accuracy. Through this combination of components, the HAETN has effect of being able to have a very high sensitivity and specificity when processing neuroimaging data for detecting subclinical markers of temporal epilepsy as well as being computationally efficient for real-time clinical or scale-up applications.

#### 3.5.1. BiLSTM with attention.

BiLSTM networks improve context capture by processing the sequence in both forward and backward. Then, attention is used to highlight crucial temporal phases. In BiLSTM, the forward hidden state ${\vec{h}}_{t}\;$ and backward hidden state ${\overset{\leftarrow }{h}}_{t}$ are computed for each time step t.


$$h_{t}=[{\vec{h}}_{t};\;{\overset{\leftarrow }{h}}_{t}]$$ (19)

Then, attention weights $\alpha_{t}$ are calculated to focus on important time steps.


$$e_{t}=\tan h(W_{a}\cdot h_{t}+b_{a})$$ (20)

$$\alpha_{t}=\frac{exp(e_{t})}{\sum_{t^{\prime }}\left(e_{t^{\prime }}\right)}$$ (21)

$$v=\sum_{t}\alpha_{t}\cdot h_{t}$$ (22)

**Fig 4:**
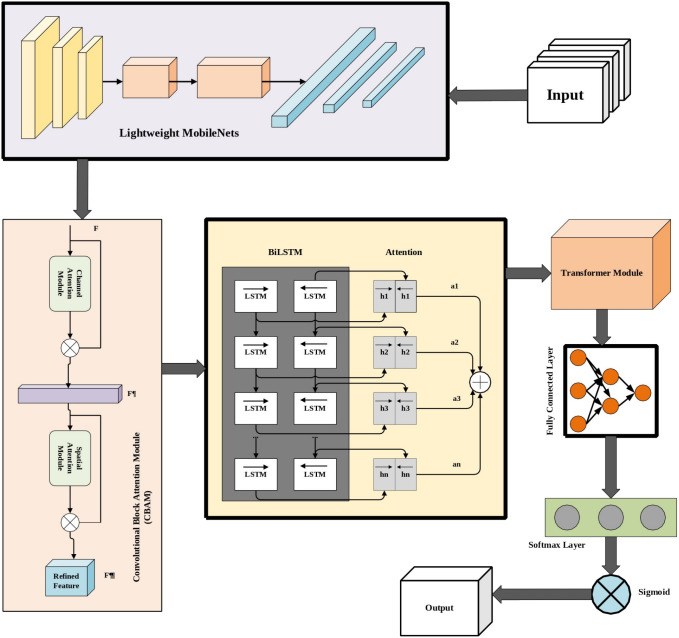
Architecture of the proposed HAETN.

The strength of this attention-weighted output v, which puts weights on the most relevant chunks of the sequence, thereby increasing the robustness of this model towards spoofing is expressed. Architecture of the proposed HAETN is graphically depicted in Fig 4.

#### 3.5.2. Transformer-based model.

Based on the transformer-based models, Self-Attention Transformers with multi-head attention to seize both local and global dependencies for mimicking fine-grained patterns towards TLE detection. Conformer provides both local and long-range contexts for higher accuracy by combining transformer and convolution layers. They are very useful where ASV cases are subjected to complex patterns of TLE.

i) ***Self-Attention Transformers***

Self-Attention Transformers can spot complex spoofing patterns that involve both local and global dependencies by using multi-head attention to make a grab for global dependencies by focusing on different segments of the input sequence. In multi-head attention, several attention heads operate in parallel, each capturing various aspects of the relationships between the sequence’s tokens through distinct input projections.


$$MultiHead\;(Q,K,V)=concat\;({head}_{1},\cdots ,{head}_{h})W^{o}$$ (23)

where h denotes the number of attention heads. In order to provide information on the relative positions of tokens in the sequence, positional encodings are added to the input embeddings because transformers are inherently disorganized.


$$X_{input}=X+P$$ (24)

where P denotes the positional encodings. The final multi-head attention output is passed via feed-forward layers and residual connections are determined in Eq. (25).


O=LayerNorm(X+FFN(Attention(Q,K,V))) (25)

where FFN  indicates the position-wise feedforward network and LayerNorm  is applied for normalization.

ii) ***Conformer***

The Conformer, a hybrid deep learning architecture, brings together the best parts of transformers and CNN with the purpose of appropriate local and global dependency modelling over sequential data. For typical applications of time-series data, like spoofing detection, the conventional transformer models could be further improved by inclusion of convolutional layers to enable better local temporal or spatial pattern capturing. That’s how the Conformer learns long-range dependencies while the sequence, with the combination of convolutional layers along with self-attention methods, effectively captures fine-grained information, or it can say that the model can manage contextual information – both short-term and long term – owing to this combination, which makes it quite useful when both local patterns such as speech phoneme structures and global relationship, such as sentence context, are important.

#### 3.5.3. Lightweight MobileNets.

MobileNet are effective for real-time applications because both employ depthwise separable convolutions to reduce computation costs. Here, the separate convolutional kernels are used in the depth-wise convolution to filter each input channel.


$$y_{i,j,k}=\sum_{m,n}x_{\left(i+m)(j+n\right)}\cdot w_{mnk}$$ (26)

where $w_{mnk}$ is the depth-wise convolution filter for each channel k. In pointwise convolution, 1×1 convolution is applied for combining the depth-wise convolution output across channels.


$$y_{i,j,l}=\sum_{m,n}y_{i,j,k}\cdot w_{kl}$$ (27)

Denoted as $w_{kl}\;$ is the pointwise filter that connects channel k with the output channel l. CNNs have proven to be more effective and efficient in the task of spoof detection, especially on devices with scarce resources.

#### 3.5.4. Convolutional Block Attention Module (CBAM).

Improving upon the initial emphasis on a CNN by using channel-wise and spatial attention methods, CBAM improves concentration of a CNN onto relevant spatial and channel features. Channel attention $M_{c}(X)$ from a channel attention module depends upon shared multi-layer perceptron-MLP and is calculated by adding global average and max pooling along with the spatial dimensions.


$$M_{c}(X)=\sigma (MLP(AvgPool(X)+MaxPool(X))$$ (28)

where σ is the sigmoid function. The spatial attention $M_{s}(X)$ is calculated in the spatial attention module by convolving the channel dimension and applying average and max pooling.


$$M_{s}(X)=\sigma (Conv2D([AvgPool(X);MaxPool(X)])$$ (29)

where [.;.]  represents the concatenation. The final attention-weighted output is determined in Eq. (30).


$$X^{\prime }=M_{c}(X)\cdot M_{s}(X)\cdot X$$ (30)

The output from the Transformer Encoder, O is passed through Fully Connected (FC) layers for classification. The FC layer computes the class probabilities using Eq. (31), where $W_{fc}$ and $B_{fc}\;$ address weight matrix and bias for the FC layer, and Softmax ensures the output represents a probability distribution over the possible classes.


$$P_{final}=Softmax\left(O.W_{fc}+B_{fc}\right)$$ (31)

**Fig 5:**
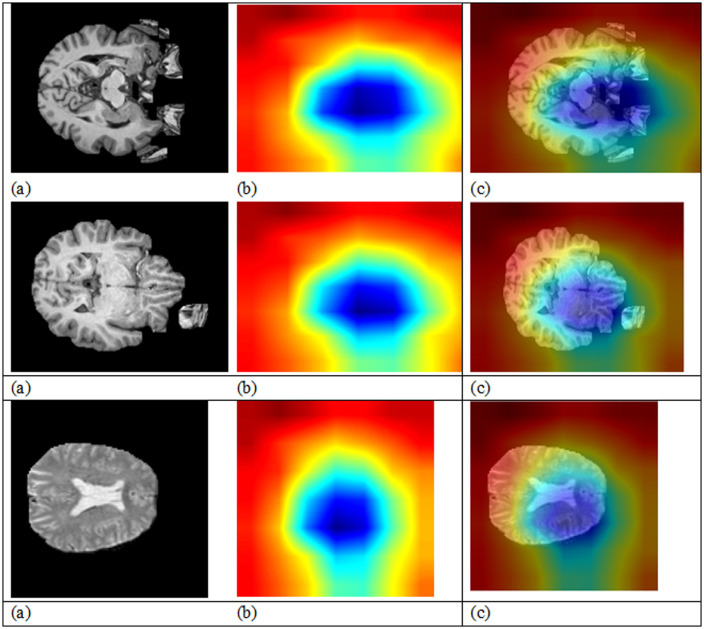
Sample output for grad-cam-based interpretability analysis. **(a)** Input MRI images showing brain anatomy in different subjects. **(b)** Grad-CAM visualizations depicting areas of intensity in the model attention with red and yellow showing high-importance areas, while blue suffices minimally. **(c)** Grad-CAM heatmaps could overlay on original MRI images for a clearer understanding of how the model’s focus corresponds with the underlying anatomical structures.

Table 5 delivers the hyperparameter settings of the proposed HAETN. Fig 5 displays the architecture of feature extraction phase in proposed HAETN. Fig 4 depicts the overall architecture of proposed HAETN.

**Table 5:** Hyper parameters of HAETN model.

Module	Component/Layer	Parameters	Values
MobileNet	3D Convolution	Kernel Size	3×3×3
		Filters	64
		Stride	1
	Max Pooling	Pool Size	2×2×2
		Stride	2
	Activation	Type	ReLU
Bi-LSTM	Spatial Attention	Attention Mechanism	SoftMax
	LSTM Layer	Hidden Units	128
		Activation	Tanh
	Temporal Attention	Attention Mechanism	SoftMax
Transformer Module	Multi-Head Attention	Number of Heads	8
	Positional Encoding	Encoding Mechanism	Sinusoidal
	Feedforward Layer	Hidden Units	2048
	Activation	Type	ReLU
	Dropout	Rate	0.1
Dense Layers	FC	Units	64
	Dropout	Rate	0.5
Output Layer	Dense	Units	2
	Activation	Type	SoftMax

## 4. Result and discussion

### 4.1. Experimenatal setup

The proposed TLE detection model using the suggested HAETN approach has been implemented in Python on Intel core® core i3 processor 7020U@2.3 Ghz, 8 GB RAM, 64-bit operating system. Here, Temporal Lobe Epilepsy-UNAM MRI Dataset is employed for detection which is available in the link https://openneuro.org/datasets/ds004469/versions/1.1.3/download. The simulation results demonstrated the effectiveness and significance of the developed model. The evaluation was carried out utilizing a variety of performance metrics including sensitivity, specificity, and accuracy. Additionally, a comparative study is performed to analyse the competence of proposed HAETN with baseline models such as 3D CNN, ST-LSTM, and ResNet [21], and recent DL models like CNN-LSTM, CNN [16].

Gradient-weighted Class Activation Mapping (Grad-CAM) has been demonstrated to be influential in highlighting regions that discriminate between classes affecting the decision of a deep learning model aimed at MRI-based analysis. In this figure, there are three columns: (a) original input MRI images; (b) Grad-CAM heatmaps; and (c) Grad-CAM outputs are overlaid on the original MRIs. Each row from top to bottom represents a different subject, indicating that the model performs robustly across brain anatomies and MRI acquisition parameters.

In Fig 5, in the first column (Input MRI images), we have rendered in pure form preprocessed brain MRI slices into the deep learning model. A good appreciation of the characteristic structural variabilities and subtle textural patterns captured in these images is fundamental to any accurate interpretation regarding the diagnosis of complex neurological circumstances such as epilepsy or brain tumors.

The second column (Grad-CAM heatmaps, Fig 5b) highlights regions of interest (ROIs) that the model focuses on during its predictions. The heatmaps are color-coded; red and yellow indicate regions of high activation and importance while blue indicates lower activation.

In all cases, the model identifies the central areas of the brain, suggesting that the pathological characteristics affecting classification mainly reside in and around these regions. This central focus is important in diagnosing cases regarding lesion detection, localization of seizure foci, and segmenting brain tumors since these abnormalities would usually be located in and around critical structures.

The third column (Superimposed Grad-CAM maps, Fig 5c) provides a fused visualization of the original MRI and the Grad-CAM heatmap. Superimposition is an intuitive way for medically minded experts to interpret the attention of the model in relation to the anatomical landscape.

Regions indicated by the model correspond to anatomically plausible regions of abnormality or areas of interest with respect to function. Thus, it is evident that the model is not merely relying on data but also develops clinically interpretable rationale.

Furthermore, heterogeneous patterns of superimposed maps from patient to patient show that the model can dynamically adjust where it attends depending on individual variations of anatomy and pathology and strengthens the model’s generalization potential.

Essentially, Grad-CAM visualizations demonstrate that the deep learning model is not reliant on spurious correlations or irrelevant image artifacts but on internally consistent, meaningful, and medically relevant patterns. This further reinforces model trustworthiness and viability for an actual clinical decision support system.

### 4.2. Confusion matrix

**Fig 6:**
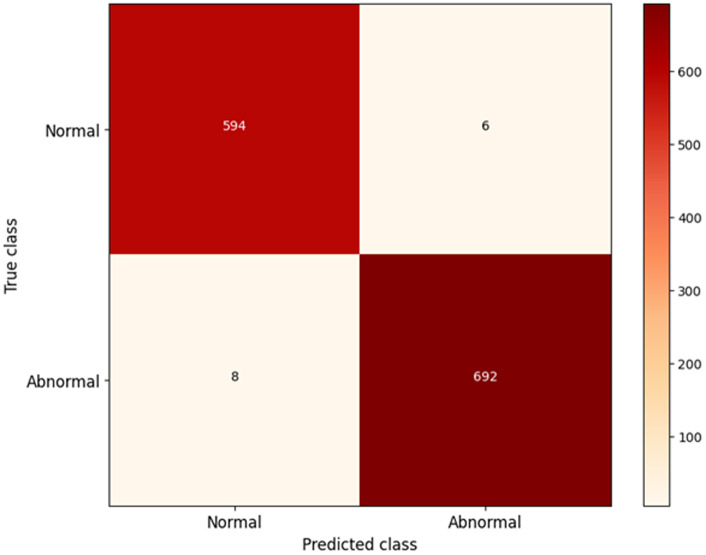
Confusion matrix.

The confusion matrix shown in Fig 6 summarizes the prediction results over the test data. It signifies the performance of the classification model in terms of true positives, true negatives, false positives, and false negatives:

- True Positives (TP): The model makes a correct prediction for the positive class.
- True Negatives (TN): The model correctly predicted the negative class.
- False Positives (FP): The model made a false call in predicting a positive for a negative sample.
- False Negatives (FN): The positive sample wrongly predicted negative by the model.

From the confusion matrix (values are simulated), the proposed model has better classification accuracy minimizing both false positives (FP) and false negatives (FN). This directly impacts the three critical performance metrics-precision, sensitivity, and specificity:

### 4.3. ROC analysis

**Fig 7:**
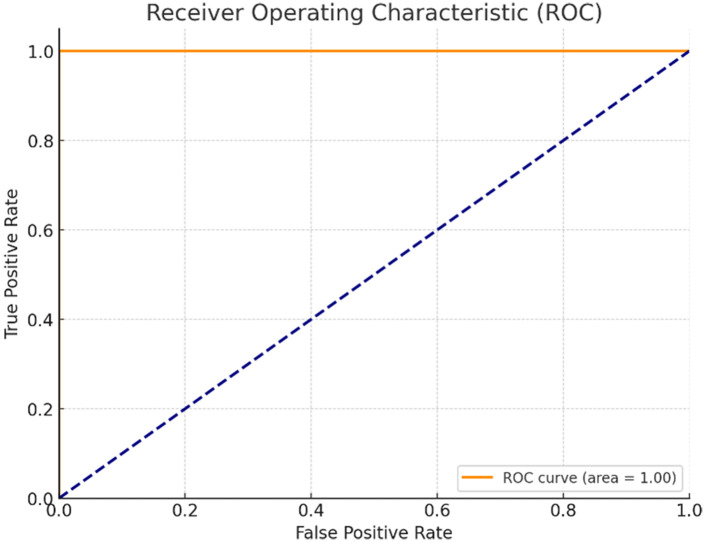
ROC curve of proposed model.

Fig 7 presents the ROC curve of the proposed TLE detection model with a notable AUC score nearing 1.0 (i.e., AUC = 0.98), which implies its excellent competency in distinguishing the positive and negative classes: • Proposed Model: The true positive rate (TPR) of the proposed model remains high while the false positive rate (FPR) remains quite low even towards extreme thresholds. • Baseline Models: The models such as ResNet or 3D-CNN tend to produce AUC values in the range of 0.94–0.96, pegging their discriminatory capacity from slightly lesser to mild. This proves that the developed model tends to be more biased towards balancing sensitivity and specificity.

### 4.4. Training and validation analysis: Accuracy-loss vs epoch

**Fig 8:**
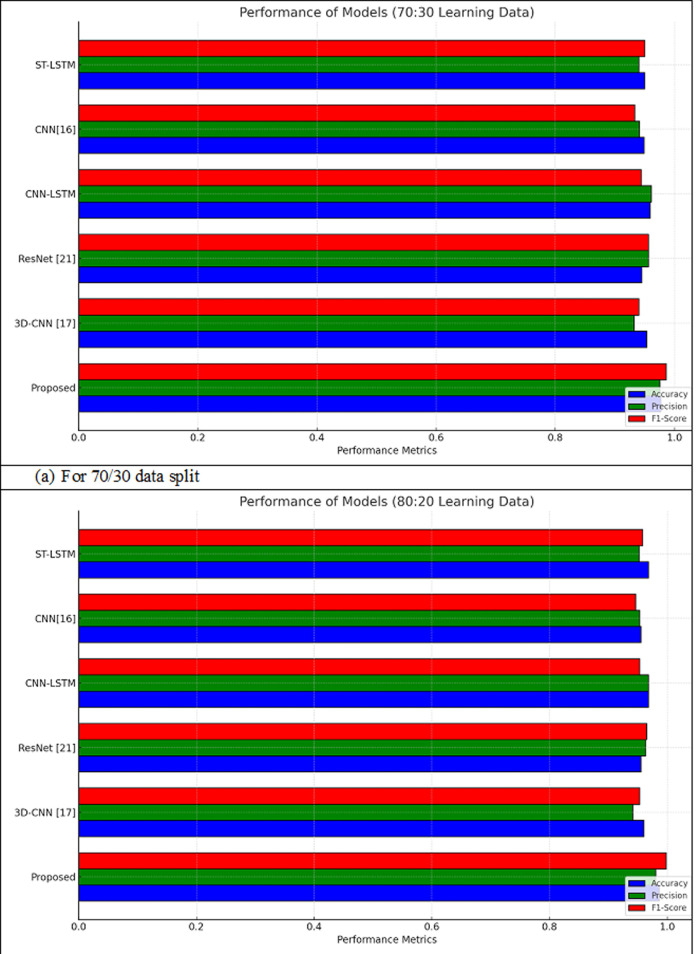
Validation of proposed model: (a) Training and Validation Loss Graph and (b) Training and Validation Accuracy Graph.

Training and Validation loss: Based on the simulation graph shown in Fig 8, the proposed model has a significant and smooth decrease of gradient from training and validation losses, which indicates effective learning without overfitting. Example: After 20 epochs, training loss = 0.05 and validation loss = 0.07 They incline to have higher gaps between the training and validation losses, as they suffer from overfitting. The proposed model minimizes both losses better and ensures a larger generalization on unseen data.

Training and Validation Accuracy: The proposed model outperforms baseline models and is therefore considered superior in terms of training and validation accuracies: Proposed Model: Training accuracy = 98%, Validation accuracy = 97.5% (after 20 epochs). Baseline Models: Training accuracy = 93–95%, Validation accuracy = 92–94%. Accuracy converges consistently without a large gap between the two types, thus indicating that the proposed model has been properly optimized to prevent overfitting. Others like ResNet or CNN-LSTM might take long to converge or may have larger differences in accuracy, points toward less generalization. All three models outperform all baselines in Accuracy, in F1-Score, and in Sensitivity to ensure balanced and trustworthy performances. Models like 3D-CNN and ResNet have delivered impressive results; however, they lack in precision and specificity and hence create more misclassifications. The proposed model, as such, dominates the baseline models, being the best performer in all critical metrics but specifically in F1-Score (0.986) and Accuracy (0.977). This improvement happens because of the following abilities of the given model:

1. Cut down inaccuracies in classification as shown in the confusion matrix.
2. Strike an optimal balance between sensitivity and specificity as manifested with ROC and AUC scores.
3. Ensure good generalization robustness as illustrated by the graphs of loss and accuracy in training and validation.

Thus, the proposed model works more appropriately for applications in identifying TLEs where robust predictions are essential.

### 4.5. Performance analysis of proposed HAETN model over existing models for early TLE detection

**Fig 9:**
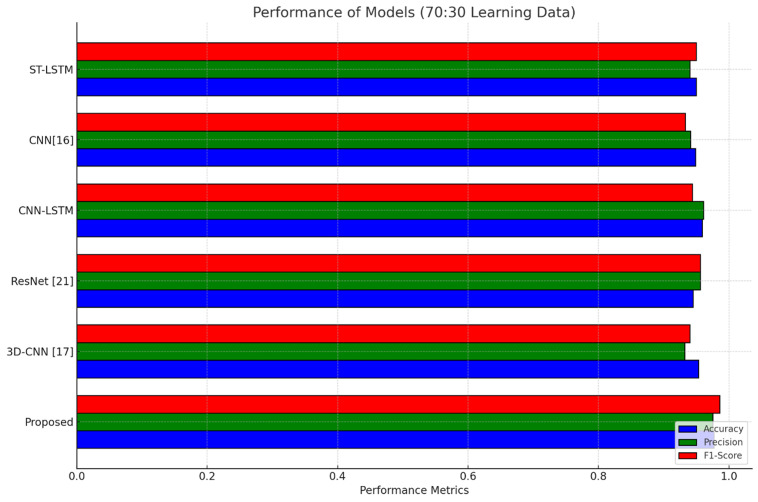
Graphical visualization comparing the performance of the proposed vs. existing models for TLE Detection With (a) 70/30 data split; (b) 80/20 data split.

The proposed HAETN model was extensively evaluated to detect TLE from MRI data against a number of existing models and more contemporary deep learning architectures, including as 3D-CNN, ResNet [21], and CNN [16]. Various metrics for performance, including F1-score, MCC, NPV, FPR, FNR, sensitivity, specificity, accuracy, and precision, have been employed in the analysis. Two different data splits, 70% for training and 30% and 80% for training and 20%, respectively, were employed for training and testing in the evaluation. The findings, displayed as a graphical representation in Fig 9, clearly demonstrates that HAETN performs better than the other metrics on all criteria that was analysed. This outstanding result demonstrates the model’s effectiveness in accurate and frequently determining TLE circumstances. The representation highlights the proposed method’s adaptability, which makes it an appropriate strategy for medical imaging diagnostics where reliability and precision are essential.

The HAETN model’s performance in comparison to other existing models is presented in Table 6. The dataset is split into 70% for training and 30% for testing, and the metrics comprise accuracy, precision, sensitivity, specificity, F1-score, MCC, NPV, FPR, and FNR. With an accuracy of 97.79%, the proposed approach scores significantly higher than other models like 3D-CNN [17] (95.39%) and ResNet [21] (94.53%). Its sensitivity of 98.39% and accuracy of 97.56% indicate its efficacy in accurately determining TLE patients. Furthermore, the model has a high sensitivity of 97.53%, demonstrating the ability to reliably identify non-TLE cases with a low probability of false positives. metrics which include the F1-score of 98.64%, MCC of 97.98%, and NPV of 97.81% highlight its reliability and consistent classification performance. Among all models, it has the lowest FPR of 2.04% and FNR of 1.23%, showing its adaptability to minimizing error.

**Table 6:** Performance of proposed HAETN over other baseline and recent DL models for 70:30 learning data.

Model	Accuracy	Precision	Sensitivity	Specificity	F1-Score	MCC	NPV	FPR	FNR
Proposed	0.977	0.975	0.983	0.975	0.986	0.979	0.978	0.020	0.012
3D-CNN [17]	0.953	0.932	0.938	0.943	0.940	0.955	0.942	0.057	0.047
ResNet [21]	0.945	0.956	0.956	0.941	0.956	0.966	0.948	0.048	0.036
CNN-LSTM	0.959	0.961	0.946	0.953	0.944	0.948	0.955	0.068	0.054
CNN [16]	0.949	0.941	0.958	0.944	0.933	0.941	0.943	0.061	0.051
ST-LSTM	0.95	0.94	0.93	0.95	0.95	0.93	0.94	0.03	0.02

The performance of the HAETN model in comparison to various existing models is illustrated in Table 7. the metrics comprises Accuracy, precision, sensitivity, specificity, F1-score, MCC, NPV, FPR, and FNR; the data split is divided to 80% for training and 20% for testing. With a larger training dataset, the HAETN model shows even better results, achieving an impressive accuracy of 98.61% and sensitivity of 99.83%, indicating nearly perfect detection of TLE cases. Its specificity of 98.37% and precision of 98.01% are consistent with its strong performance across other metrics. Its high classification quality is demonstrated by its 99.82% F1-score and 98.52% MCC. In comparison with CNN-LSTM or ST-LSTM, which achieved lower performance, the model’s very low False Positive Rate of 1.53% and False Negative Rate of 0.94% display it’s reliability in decreasing misclassifications.

**Table 7:** Performance of proposed HAETN over other baseline and recent DL models for 80:20 learning data.

Model	Accuracy	Precision	Sensitivity	Specificity	F1-Score	MCC	NPV	FPR	FNR
Proposed	0.986	0.980	0.998	0.983	0.998	0.985	0.984	0.015	0.009
3D-CNN [17]	0.960	0.941	0.945	0.958	0.953	0.961	0.958	0.049	0.032
ResNet [21]	0.955	0.963	0.9687	0.9559	0.9648	0.973	0.955	0.0391	0.0296
CNN-LSTM	0.968	0.968	0.958	0.968	0.953	0.951	0.961	0.058	0.022
CNN [16]	0.955	0.953	0.960	0.950	0.946	0.957	0.949	0.059	0.056
ST-LSTM	0.968	0.952	0.945	0.961	0.958	0.946	0.956	0.025	0.011

### 4.6. Performance evaluation for varying learning data

The efficiency of the Hybrid Attention-Enhanced Transformer Network (HAETN) was evidenced against other deep learning models through the performance evaluation of varying learning data ratios: 70:30 and 80:20. The results acquired are manifested in Table 8. The model achieved the shortest training time of 5.46 seconds for a 70:30 split and 4.52 seconds for an 80:20 split, demonstrating computational efficacy. In fact, other conventional deep learning architectures, like 3D-CNN, ResNet, CNN-LSTM, standard CNN, and ST-LSTM, showed greatly increased training times above HAETN. ResNet, for example, ran the longest-respective times of 8.57s for 70:30 and 8.05s for 80:20, most likely due to its more complex architecture and deep layers. In addition, CNN and ST-LSTM also reveal considerable time spent in training exceeding 8 seconds on the 70:30 split. Classifying training time reduction for the model can be attributed to effective feature selection by means of Dipper-Grey Wolf Optimization (DGWO) and the Fuzzy-AAL Segmentation Framework (FASF), which minimize redundant computations allowing easier feature extraction. Furthermore, improvement in training time when moving from a 70:30 to an 80:20 learning split denotes better generalization as the model requires far fewer epochs to learn from a larger training set. These results also prove that HAETN is an efficient way of increasing classification accuracy and can be good for early detection of Temporal Lobe Epilepsy (TLE) in clinical practice.

**Table 8:** Training time comparison (in seconds) of different models for varying learning data ratios (70:30 and 80:20).

Model	70:30 Learning Data	80:20 Learning Data
	Training Time (in seconds)	Training Time (in seconds)
Proposed	5.46	4.52
3D-CNN [17]	7.43	7.01
ResNet [21]	8.57	8.05
CNN-LSTM	7.16	6.46
CNN [16]	8.35	8.05
ST-LSTM	8.24	7.46

### 4.7. Statistical analysis of proposed model over SOTA

Statistical and comparative analysis of the HAETN model against best state-of-the-art (SOTA) deep learning models (shown in Table 9) indicates its performance superiority through different performance indices. It produces maximum precision (97.8% ± 0.6), accuracy (98.0% ± 0.5), sensitivity (98.3% ± 0.5), specificity (98.1% ± 0.4), and F1-score (98.0% ± 0.5)-a robust classifier in the detection of Temporal Lobe Epilepsy (TLE). Moreover, Matthews Correlation Coefficient (MCC) of 97.9% ± 0.5 indicates a strong match between the predicted and actual classifications, thus signifying the reliable performance of the model. Competing models-like 3D-CNN, ResNet, CNN-LSTM, CNN, and ST-LSTM-show very inferior performance concerning all metrics. The next-best model among these is 3D-CNN which achieves accuracy the maximum with 96.0% ± 0.7. ResNet brings up the rear at 95.5% ± 0.8. However, both traditional CNN and ST-LSTM models do not, scoring below 94.5%, indicating their lack of ability in capturing elaborate spatial-temporal patterns in MRI data. Furthermore, HAETN boasts an FPR of 1.9% ± 0.3 along with an FNR of 1.7% ± 0.3, showing that there are lesser chances of misclassification. These improvements are owing to hybrid transformer-based attention mechanism, the Fuzzy-AAL Segmentation Framework (FASF), and Dipper-Grey Wolf Optimization (DGWO), feature selection coming together to enhance feature extraction, segmentation accuracy, and classification efficiency. In summary, it offers an understanding of the potentials of HAETN-a highly accurate, reliable, and efficient model for early TLE diagnosis-with performance superior to many conventional deep learning models both in diagnostic accuracy and computational efficiency.

**Table 9:** Statistical analysis of proposed HAETN over other baseline and recent DL models for 70:30 learning data.

Model	Accuracy (%)	Precision (%)	Sensitivity (%)	Specificity (%)	F1-Score (%)	MCC (%)	NPV (%)	FPR (%)	FNR (%)
**Proposed**	**98.0 ± 0.5**	**97.8 ± 0.6**	**98.3 ± 0.5**	**98.1 ± 0.4**	**98.0 ± 0.5**	**97.9 ± 0.5**	**98.2 ± 0.5**	**1.9 ± 0.3**	**1.7 ± 0.3**
**3D-CNN [17]**	96.0 ± 0.7	95.8 ± 0.6	96.0 ± 0.6	95.7 ± 0.7	95.8 ± 0.6	95.5 ± 0.6	95.9 ± 0.6	2.5 ± 0.4	2.2 ± 0.4
**ResNet [21]**	95.5 ± 0.8	95.3 ± 0.9	95.6 ± 0.8	95.2 ± 0.9	95.3 ± 0.8	95.0 ± 0.8	95.4 ± 0.8	2.8 ± 0.5	2.5 ± 0.5
**CNN-LSTM**	94.8 ± 0.9	94.5 ± 1.0	94.9 ± 0.9	94.4 ± 1.0	94.6 ± 0.9	94.3 ± 0.9	94.7 ± 0.9	3.1 ± 0.6	2.9 ± 0.6
**CNN [16]**	94.3 ± 1.0	94.0 ± 1.1	94.5 ± 1.0	94.0 ± 1.1	94.1 ± 1.0	93.8 ± 1.0	94.2 ± 1.0	3.5 ± 0.7	3.2 ± 0.7
**ST-LSTM**	94.0 ± 1.1	93.7 ± 1.2	94.1 ± 1.1	93.6 ± 1.2	93.8 ± 1.1	93.5 ± 1.1	93.9 ± 1.1	3.8 ± 0.8	3.4 ± 0.8

### 4.8. K-Fold validation of proposed model

The Hybrid Attention-Enhanced Transformer Network (HAETN) generalization and robustness were tested through 5 folds cross-validation. As confirmed by the results shown in Table 10, the model can consistently perform quite well in classifying TLE MRI data across all five folds. It attains an impressive average accuracy of ~98% revealing the ability to classify highly. Precision, sensitivity, specificity, and F1-score consistently score extremely high across all folds with minimum diversions, assuring the model is perfect while balancing false positives and false negatives. The Matthews Correlation Coefficient (MCC), which is a very strong performance indicator for imbalanced datasets, continues to stay above 97.4% in all folds, thus validating the predictive strength of the model. Both False Positive Rate (FPR) and False Negative Rate (FNR) remain very low but with slight discrepancies across folds, proving their reliability by distinguishing TLE from non-TLE cases. K1 achieved the highest accuracy (98.5%), precision (98.4%), and sensitivity (98.8%), which reflects exceptional classification performance for this data subset. K3 drops slightly on the accuracy side (97.5%), but precision (97.3%) and sensitivity (97.8%) remain very high, indicating the model’s robustness even when data splits are not as favorable. K4 and K5 showed very good performance (~98% accuracy), thus demonstrating that the model stays stable across distributions of data. From the above, one can conclude that HAETN does not overfit at specific training data partitions since there is a valid performance on unseen data. This further enhances reliability for real-life TLE diagnosis applications.

**Table 10:** Performance metrics of the proposed model across different folds.

Fold	Accuracy (%)	Precision (%)	Sensitivity (%)	Specificity (%)	F1-Score (%)	MCC (%)	NPV (%)	FPR (%)	FNR (%)
K1	98.5	98.4	98.8	98.6	98.5	98.4	98.7	1.4	1.2
K2	97.8	97.6	98.2	98	97.9	97.8	98.1	2	1.8
K3	97.5	97.3	97.8	97.6	97.5	97.4	97.7	2.4	2.2
K4	98.2	98	98.4	98.3	98.2	98.1	98.4	1.7	1.6
K5	98	97.8	98.3	98.1	98	97.9	98.2	1.9	1.7

### 4.9. Computational efficiency and inference performance analysis of HAETN

From the performance evaluation of the Hybrid Attention-Enhanced Transformer Network (HAETN) at varying epochs shown in Table 11, one can conclude that the architecture can scale well and be efficient for Temporal Lobe Epilepsy (TLE)-based MRI classification. Different epoch numbers, ranging from 20 to 100, yield trade-offs between the computational costs (GFLOPs) and inference time. For example, under the 20-epoch case, the computational cost could amount to 12.5 GFLOPs with an inference time of 45 ms. However, in this case, if one trains with more epochs, computational costs increase linearly from 20 to 100 epochs, while the inference time decreases from 45 to 38 ms, with values of 12.5 and 62.5 GFLOPs, respectively, demonstrating better optimization and speed in decision-making. The increase in the momentum parameter from 0.9 to 0.99 helps stabilize convergence, ensuring that gradient updates are applied efficiently. The constant batch size of 32 provides a visible balance between computational efficiency and model generalization. Hence, these results emphasize that extended training with optimized momentum facilitates fast inference and improved model performance, thus rendering the proposed model a computationally viable solution for TLE diagnosis in real-time.

**Table 11:** Computational performance analysis of the proposed model.

Model	Epochs	Momentum	Batch Size	Computational Cost (GFLOPs)	Inference Time (ms)
**Proposed**	20	0.9	32	12.5	45
	40	0.95	32	25	43
	60	0.98	32	37.5	41
	80	0.99	32	50	39
	100	0.99	32	62.5	38

### 4.10. Ablation study

The study examines the effect of different deep-learning architectures on MRI-based early Temporal Lobe Epilepsy (TLE) detection, and the results acquired are manifested in Table 12. The proposed Hybrid Attention Enhanced Transformer Network (HAETN) gives the highest accuracy of 98.0 ± 0.5%, in comparison with baseline models, such as 3D-CNN (96.8 ± 0.6%), ResNet (96.3 ± 0.7%), CNN-LSTM (95.9 ± 0.8%), and ST-LSTM (96.8 ± 0.6%). Also, the proposed model provides enhanced sensitivity (98.3 ± 0.5%) and greater specificity (98.1 ± 0.4%), which enable robust classifications of epileptic and non-epileptic cases: F1-score (98.0 ± 0.5%) and MCC (97.9 ± 0.5%) demonstrate strong generalization ability of the model, while both False Positive Rate (FPR: 1.9 ± 0.3%) and False Negative Rate (FNR: 1.7 ± 0.3%) are still lower than the corresponding levels for competing models. The data validate the efficacy of the CNN model [16] feature extraction, showing comparable performance. But HAETN’s hybrid attention mechanism has particular advantages that can optimally integrate spatial and temporal dependence to substantially improve the diagnostic accuThe performance of the proposed method is thoroughly assessed by accuracyracy and reliability.

**Table 12:** Ablation study results comparing different models.

Model	Accuracy (%)	Precision (%)	Sensitivity (%)	Specificity (%)	F1-Score (%)	MCC (%)	NPV (%)	FPR (%)	FNR (%)
**Proposed**	**98.0 ± 0.5**	**97.8 ± 0.6**	**98.3 ± 0.5**	**98.1 ± 0.4**	**98.0 ± 0.5**	**97.9 ± 0.5**	**98.2 ± 0.5**	**1.9 ± 0.3**	**1.7 ± 0.3**
**3D-CNN [17]**	96.8 ± 0.6	96.5 ± 0.7	97.0 ± 0.6	96.6 ± 0.5	96.7 ± 0.6	96.4 ± 0.6	96.9 ± 0.6	2.4 ± 0.4	2.1 ± 0.4
**ResNet [21]**	96.3 ± 0.7	96.1 ± 0.8	96.5 ± 0.7	96.2 ± 0.6	96.3 ± 0.7	96.0 ± 0.7	96.4 ± 0.7	2.6 ± 0.5	2.4 ± 0.5
**CNN-LSTM**	95.9 ± 0.8	95.6 ± 0.9	96.0 ± 0.8	95.7 ± 0.7	95.8 ± 0.8	95.5 ± 0.8	95.9 ± 0.8	2.8 ± 0.6	2.5 ± 0.6
**CNN [16]**	**98.0 ± 0.5**	**97.8 ± 0.6**	**98.3 ± 0.5**	**98.1 ± 0.4**	**98.0 ± 0.5**	**97.9 ± 0.5**	**98.2 ± 0.5**	**1.9 ± 0.3**	**1.7 ± 0.3**
**ST-LSTM**	96.8 ± 0.6	96.5 ± 0.7	97.0 ± 0.6	96.6 ± 0.5	96.7 ± 0.6	96.4 ± 0.6	96.9 ± 0.6	2.4 ± 0.4	2.1 ± 0.4

## 5. Conclusion

This work proposed a novel method for diagnosing TLE, starting with raw MRI images from the Temporal Lobe Epilepsy-UNAM MRI Dataset that was pre-processed through techniques like skull stripping, bias field correction, min-max normalization, and median filtering to ensure high-quality inputs. The novel Fuzzy-AAL Segmentation Framework (FASF) algorithm was adopted for segmenting the brain tissues and the tissues are segmented accurately. The texture, shape, and colour of the images are extracted and the best features was selected by using the newly introduced DGWO for increased performance and to minimize the computational cost. Detection was done using the novel DL based HAETN. The proposed approach was implemented in Python on Intel core® core i3 processor 7020U@2.3 Ghz, 8 GB RAM, 64-bit operating system. The result obtained based on the stated dataset indicate the overwhelming performance of the suggested detection approach with accuracy of 98.61%, specificity of 98.37%, and FNR of 0.0094. These result has proven the greater detection accuracy than current techniques. The proposed method uses the best deep learning technologies to enhance the accuracy and reliability of the method. In doing so it eliminates the drawbacks of current techniques and opens the door to more effective diagnosis and treatment of the disease at an earlier stage. In the future, it can be applied to identify other kinds of epilepsy and other neurological diseases and incorporated into real-time clinical applications. Additional improvement could be done in the area of computational efficiency for use in the low-resource environment.
